# Bacteriological quality of bottled drinking water versus municipal tap water in Dharan municipality, Nepal

**DOI:** 10.1186/s41043-016-0054-0

**Published:** 2016-06-07

**Authors:** Narayan Dutt Pant, Nimesh Poudyal, Shyamal Kumar Bhattacharya

**Affiliations:** 1 Department of Microbiology, Grande International Hospital, Dhapasi Kathmandu, Nepal; 2 Department of Microbiology, B. P. Koirala Institute of Health Sciences, Dharan, Nepal

**Keywords:** Bacteriological quality, Fecal coliform count, Fecal streptococcal count, Total plate count, Total coliform count

## Abstract

**Background:**

Water-related diseases are of great concern in developing countries like Nepal. Every year, there are countless morbidity and mortality due to the consumption of unsafe drinking water. Recently, there have been increased uses of bottled drinking water in an assumption that the bottled water is safer than the tap water and its use will help to protect from water-related diseases. So, the main objective of this study was to analyze the bacteriological quality of bottled drinking water and that of municipal tap water.

**Methods:**

A total of 100 samples (76 tap water and 24 bottled water) were analyzed for bacteriological quality and pH. The methods used were spread plate method for total plate count (TPC) and membrane filter method for total coliform count (TCC), fecal coliform count (FCC), and fecal streptococcal count (FSC). pH meter was used for measuring pH.

**Results:**

One hundred percent of the tap water samples and 87.5 % of the bottled water samples were found to be contaminated with heterotrophic bacteria. Of the tap water samples, 55.3 % were positive for total coliforms, compared with 25 % of the bottled water. No bottled water samples were positive for fecal coliforms and fecal streptococci, in contrast to 21.1 % and 14.5 % of the tap water samples being contaminated with fecal coliforms and fecal streptococci, respectively. One hundred percent of the tap water samples and 54.2 % of the bottled water samples had pH in the acceptable range.

**Conclusions:**

All of the municipal tap water samples and most of the bottled drinking water samples distributed in Dharan municipality were found to be contaminated with one or more than one type of indicator organisms. On the basis of our findings, we may conclude that comparatively, the bottled drinking water may have been safer (than tap water) to drink.

## Background

The quality of drinking water is of great concern to mankind, but drinking water supplies have a long history of being contaminated by a wide spectrum of microbes including the fecal coliforms [1]. Contaminated water can cause a spectrum of diseases ranging from self-limiting gastrointestinal disturbances to severe life-threatening infections [2]. According to World Health Organization (WHO), 80 % of the diseases in developing countries are either water or sanitation related [3].

Recently, there has been a considerable worldwide increase in the consumption of bottled water due to consumer’s awareness regarding bottled water as a healthy alternative to tap water. However, bottled water is not necessarily safer than tap water. Many studies have reported the presence of heterotrophic bacteria along with coliforms in bottled water in counts, exceeding national and international standards [4].

World Health Organization (WHO) has reported that about 30,000 people and children die everyday from water-related diseases, more critically, in developing or least developing countries. According to the data published by public health department, Nepal government, every year, about 3500 children die due to water-related illnesses [5].

So, it has become imperative to assess the quality of drinking water to ensure that if it is acceptable for human consumption. We tested the bacteriological quality and pH of the municipal tap water and bottled drinking water and compared their quality.

## Methods

### Study design

The present study was a community-based cross-sectional study conducted in the Department of Microbiology, B. P. Koirala Institute of Health Sciences (BPKIHS) from July 2011 to June 2012. Twenty-four bottled water samples and 76 tap water samples collected from Dharan municipality were tested for bacterial contamination, pH, and temperature.

#### Sample size calculation

In most of the studies conducted in developing countries including Nepal, the prevalence of bacterial contamination (due to heterotrophic bacteria) for tap water was around 95 % and that for bottled water was around 99 %. So, by using the following formula, the sample sizes for both tap water and bottled water were calculated.

$$ \mathrm{Sample}\ \mathrm{size} = {Z}^2P\left(1-P\right)/{C}^2 $$

where

*Z* = *Z* value (1.96 for 95 % confidence level)

*P* = prevalence

*C* = confidence interval (0.05)

The sample size for tap water was calculated to be 73 and that for bottled water was calculated to be 15. But, we took comparatively larger sample size.

### Water sample collection

Since this study was conducted in a low-income country with limited resources, we chose the sample size according to the availability of the resources. For the collection of tap water, the Dharan municipality was divided into 19 approximately equal parts. Within each area, a main street was identified and the samples were collected from each fifth tap on alternating sides of the street until four samples were collected, for a total of 76 samples. For tap water, two sterile bottles, each of 200-ml capacity containing sodium thiosulfate (to neutralize any chlorine if present) were used. The mouth of the tap was cleaned by using clean cloth to remove any dirt if present. Then, the sterilization of the mouth of the tap was done with the help of flame. The tap was turned on and allowed the water to run for 1–2 min at a medium flow. Sterilized bottle was opened and filled with water by leaving a small air space to make shaking before analysis easier. Finally, a stopper was placed on the bottle and a brown paper protective cover was fixed with the string. For the collection of the bottled drinking water, the numbers of registered bottled drinking water distributors in the Dharan municipality were identified. There were 8 bottled drinking water distributors distributing 8 different brands of bottled drinking water. A total of 24 bottled drinking water samples (3 samples from each brand) were collected. The basic assumptions for the sampling strategy we have followed were; almost every area of the Dharan was included and no bottled water brand present in the market of Dharan at the time was left. And the main purpose of choosing the particular sampling strategy was to include different water samples with different bacteriological qualities; as the tap water from different areas might have a very different quality due to different factors like leakage in distribution system, difference in quality of water supplied from sources or reservoir tanks, etc.

### Transport and analysis of samples

pH was measured by using pH meter and temperature was measured by using thermometer. The pH and temperature were measured at the sites of sample collection. The water samples were transported to the water bacteriology laboratory of BPKIHS in ice box within 2 hrs of collection. Analysis of the water sample was done within 6 hrs of collection. Detection of bacterial contamination in water samples was done in terms of total plate count (TPC) (by spread plate method); and total coliform count (TCC), fecal coliform count (FCC) and fecal streptococcal count (FSC) (by membrane filter method) [6–8].

### Identification of the bacterial isolates

All the bacteria grown on bile esculin agar (BEA), eosine methylene blue agar (EMB), m-endo agar les (MEA), and plate count agar (PCA) were subjected to identification. The *Pseudomonas* spp., *Acinetobacter* spp., and *Staphylococcus* spp. were detected in plate count agar. *Citrobacter* spp., *Enterobacter* spp., *Klebsiella* spp., *Serratia marcescens*, and *Chromobacterium violaceum* were detected in m-endo agar les (MEA). After filtering the water through the membrane filter, it was kept on m-endo agar les (MEA), which showed the growth of different types of colonies after 48 hrs of aerobic incubation at 37 °C, and on identification, these colonies were found to be of *Citrobacter* spp., *Enterobacter* spp., *Klebsiella* spp., *S. marcescens*, and *C. violaceum*. Similarly, *Enterococcus* spp. was detected by membrane filter technique by using BEA. The bacterial isolates were identified with the help of colony morphology, Gram’s staining, and biochemical properties. The biochemical tests used were catalase test, oxidase test, citrate utilization test, urease test, sulfide indole motility test, triple sugar iron test, methyl-red Voges Proskauer test, lysine decarboxylase test, slide coagulase test, tube coagulase test, growth on bile esculin agar at 44.5 °C, etc. In case of some bacteria like fecal coliforms, total coliforms, and fecal streptococci, their colony morphology in selective media like eosin methylene blue agar, m-endo agar les, and bile esculin agar, respectively, further helped in identification.

### Data analysis

The data obtained were entered into Microsoft (MS) excel and analyzed using Statistical Package for Social Sciences (SPSS) version 11.0. Mean and standard deviation were calculated, and according to the nature of the data, *P* value was determined by applying *T* test, Mann-Whitney *U* test, chi-square test, and Fisher’s exact test. *P* value <0.05 was taken as significant.

## Results

### Different characteristics of water samples

The temperatures of the tap water and bottled water were found to be 16.01 ± 3.62 °C and 17.75 ± 0.44 °C, respectively. Similarly, the pH of the tap water was found to be 7.63 ± 0.50 and that of the bottled water was 6.5 ± 0.57. The TPC/0.1 ml, TCC/100 ml, FCC/100 ml, and FSC/100 ml of the tap water were found to be 18.58 ± 17.70, 10.61 ± 22.49, 2.95 ± 9.26, and 3.33 ± 9.68 respectively, while TPC/0.1 ml and TCC/100 ml of the bottled water were 120.88 ± 85.82 and 19.33 ± 48.52, respectively. But, no bottled water were found to be contaminated with fecal coliforms and fecal streptococci. There were significant differences between the temperature, pH, TPC, and FCC of the tap water and those of the bottled water (*P* < 0.05) (Table 1).

**Table 1:** Different characteristics of water samples

Characteristics	Type of water		*P* value
	Tap water (mean ± SD)	Bottled water (mean ± SD)	
Temperature (°C)	16.01 ± 3.62	17.75 ± 0.44	<0.05
pH	7.63 ± 0.50	6.5 ± 0.57	<0.05
TPC/0.1 ml	18.58 ± 17.70	120.88 ± 85.82	<0.05
TCC/100 ml	10.61 ± 22.49	19.33 ± 48.52	0.102
FCC/100 ml	2.95 ± 9.26	0	<0.05
FSC/100 ml	3.33 ± 9.68	0	0.050

### Water samples unacceptable on the basis of different criteria

According to the WHO criteria for drinking water, on the basis of pH, no tap water samples were found to be unacceptable for drinking while 45.8 % of the bottled water samples were unacceptable and the difference was significant (*P* < 0.05). Similarly, on the basis of TPC, all the tap water samples and 87.5 % of the bottled water samples were unacceptable according to WHO criteria for drinking water (*P* < 0.05). On the basis of TCC, 55.3 % of the tap water samples and 25 % of the bottled water samples were found to be unacceptable and was statistically significant (*P* < 0.05). Similarly, on the basis of FCC and FSC, 21.1 % and 14.5 % of the tap water samples respectively were found to be unacceptable while no bottled water samples were unacceptable for drinking (Table 2).

**Table 2:** Water samples unacceptable on the basis of different criteria

Characteristics	Type of water		*P* value
	Tap water %	Bottle water %	
pH	0	45.8	<0.05
TPC	100	87.5	<0.05
TCC	55.3	25	<0.05
FCC	21.1	0	<0.05
FSC	14.5	0	0.062

### Percentage of water samples found to be contaminated with different bacteria

The most prevalent bacteria in tap water samples were gram-positive rods (100 %) followed by *Pseudomonas* spp. (76.3 %). Other bacteria isolated from tap water samples were *Citrobacter* spp. (36.8 %), *Acinetobacter* spp. (30.3 %), *Enterobacter* spp*.* (23.7 %), *Escherichia coli* (21.1 %), *Klebsiella* spp*.* (17.1 %), *Enterococcus* spp*.* (14.5 %), *Proteus* spp. (3.9 %), *Serratia* spp. (2.4 %), and *Staphylococcus* spp. (2.4 %). Similarly, the most prevalent bacteria in bottled water samples were *Pseudomonas* spp. and *Acinetobacter* spp. (87.5 %). Other bacteria isolated from bottled water samples were *Citrobacter* spp*.* (25 %), *C. violaceum* (12.5 %), and gram-positive rods (12.5 %) (Table 3).

**Table 3:** Percentage of water samples found to be contaminated with different bacteria

Bacteria	Tap water %	Bottle water %	*P* value
*Pseudomonas* spp*.*	76.3	87.5	0.241
*Acinetobacter* spp*.*	30.3	87.5	<0.05
Gram-positive rods (GPR)	100	12.5	<0.05
*Enterobacter* spp*.*	23.7	0	<0.05
*Citrobacter* spp*.*	36.8	25	0.286
*Klebsiella* spp*.*	17.1	0	<0.05
*Escherichia coli*	21.1	0	<0.05
*Enterococcus* spp*.*	14.5	0	0.062
*Chromobacterium violaceum*	0	12.5	<0.05
*Serratia marcescens*	2.6	0	1
*Proteus* spp*.*	3.9	0	1
*Staphylococcus* spp*.*	2.6	0	1

## Discussion

In our study, the bacteriological quality of bottled water was found to be better than that of tap water which was in agreement with the findings of Yasin et al. in Rawalpindi and Islamabad-Pakistan [9], Islam et al. in Dhaka [10], and Kassenga et al. in Tanzania [11]. The worse condition of the bacteriological quality of the tap water might be due to the ineffectiveness of the disinfection processes used for the treatment of water before distribution or distributing from contaminated sources without prior disinfection. But unlike our study, no significant difference was found in the bacteriological quality of tap water and bottled water by Mythri et al. in Karnataka India [12] and Ahmad and Bajahlan in Yanbu, Saudi Arabia [13]. The wrong practice of filling the bottle directly from tap water and sealing it without any prior treatment which is generally done by the bottled water manufacturers for financial benefit might be one of the reasons behind the same bacteriological quality of tap water and bottled water found in some countries. Further, in contrast to our findings, tap water was found to be superior by Abed and Alwakeel in Riyadh, Saudi Arabia [14], and Silva et al. in Brazil [15]. Due to storage of the already contaminated bottled water for a long time, its bacteriological quality may have further deteriorated to worse condition. And further, the government body responsible for monitoring the quality of bottled water might not be strict in the places where the bottled water was found to be more contaminated.

In our study, 87.5 % of the bottled water samples were contaminated with heterotrophic bacteria which was comparable with the findings by Kassenga [11] and El-Salam et al. [16]. One hundred percent of the bottled water samples were contaminated with heterotrophic bacteria in the study done by Majumder et al. in Bangladesh [17] and Khaniki et al. in Tehran [18]. Comparatively, lower rates of contamination were detected by Islam et al. (50 %) in Dhaka city [10] and Yasin et al. (30 %) in Rawalpindi and Islamabad-Pakistan [9]. Bhandari et al. found only 28 % of the bottled water samples not meeting the Nepal standards [5]. Similar concentrations of heterotrophic bacterial contamination in bottled water as in our study were also reported by Majumder et al. (1 to >500 cfu/ml) [17], Yarsin et al. (80 to 3000 cfu/ml) [9], and Lalumandier and Ayer (0.01 to 4900 cfu/ml) [19]. Much higher concentration (10^4^ to 10^6^ cfu/ml) was reported by Karem and Hassan [20]. The bacterial concentration in bottled water generally depends on the disinfection processes used by the factory [15]. And in bottled drinking water, bacteria may be indigenous from the natural source of water or may be introduced during processing or handling [11, 21]. Although the microbial concentration in processed water is initially low, it can develop into high level during storage [22]. The reasons for this may be due to the high level of oxygen provided to the water during processing, larger surface area provided by the container, higher temperature, and the nutrients arising in the container [23, 24]. Higher concentration of the bacteria may also occur through carriers like introduced flakes of human skin, particularly in non-ozonated and non-carbonated water [25]. Though 25 % of the bottled water samples we had tested were contaminated with total coliforms, fecal coliforms were not detected in any of the samples. Similar type of result was also found by El-Salam et al. [16]. But in contradiction, Kassenga et al. (1.3 %) [11], Yarsin et al. (10 %) [9], and Abayasekara et al. (15 %) [26] detected fecal coliforms from bottled water. In our study, 45.8 % of the bottled water had pH below the minimum level of 6.5 recommended by WHO. The higher percentage of bottled water with unacceptable pH may be due to the higher numbers of heterotrophic bacteria per milliliter we found in most of the bottled water samples. The temperature of the bottled water ranged from 17 to 18 °C, and it depends upon the temperature of the environment in which it has been stored. The bacteria isolated from bottled water in our study were *Pseudomonas* spp. (87.5 %), *Acinetobacter* spp. (87.5 %), *Citrobacter* spp. (25 %), gram-positive rods (12.5 %), and *C. violaceum* (12.5 %). The presence of different species of bacteria in supposedly bacteria-free bottled water is of high concern. Whether the species of bacteria present in the water samples are pathogenic or not, the fact that these are present, the hazards of contamination, and health risks to consumers should not be taken for granted [14]. Neither epidemiological studies nor correlation with occurrence of waterborne pathogens has provided the evidence of heterotrophic plate count (HPC) values alone being directly related to health risk [27]. However, some strains of bacterial species which are the part of heterotrophic bacteria can cause infections in immune-compromised persons [27].

The use of bottled water is only based on the assumption of purity and this can be misleading [14]. In our study, the method of purification of bottled water was found to be mentioned on labeling of bottled water as UV treatment, ozonation, reverse osmosis, and microfiltration. Although, bottled water should have a shelf life of 30 days unopened [16], most bottled water companies label showed that the water is valid for 6 to 9 months. In our study, one hundred percent of tap water samples were found to be contaminated with heterotrophic bacteria. Similar rates of contamination were also found by Islam et al. in Dhaka city [10] and Chaidez et al. in Mexico [28]. But, slightly low rates of contamination were found by Anwar (91.3 %) in Punjab [29] and Nguendo-Yongsi et al. (95 %) in Yaounde [30]. Most of the natural water sources are highly contaminated [31], and in a study done by Pant et al., most of the sources and reservoirs supplying drinking water to Dharan municipality were found to be heavily contaminated [32]. Further, bacteria may enter and colonize the distribution systems through the failure to disinfect water or maintain a proper disinfection residual, excessive network leakages and improper along with inadequate disposal of sewage [33]. In our study, 55.3 % of the tap water samples were contaminated by total coliforms which was similar to the percentage detected by Kassenga et al. (49.2 %) [11] and Chaidez et al. (46 %) [28]. But, higher percentages were detected by Rai et al. (85.7 %) in Nepal [34] and Yarsin et al. (64 %) in Pakistan [9]. Fecal coliforms were isolated from 21.1 % of the tap water samples which was in agreement with the results of Kassenga et al. (26.2 %) [11] and Chaidez et al. (28 %) [28]. Quite high percentage was found by Rai et al. (67.4 %) [34]. The concentration of heterotrophic bacteria in tap water in our study ranged from 10 to1200 cfu/ml which were higher in the study done by Yasin et al. (80–3000 cfu/ml) [9] and Chaidez et al. (1–5320 cfu/ml) [28], but less numbers were detected by Lalumandier and Ayer (0.2–2.7 cfu/ml) [19]. The difference in these results obtained from different studies might be due to the different maintenance conditions of the water distribution systems, different bacteriological quality of water of sources and reservoirs supplying drinking water, and the difference in effectiveness of the disinfection processes used.

The microorganisms isolated in our study from tap water were *Pseudomonas* spp. (76.3 %), *Acinetobacter* spp. (30.3 %), Gram-positive rod (100 %), *Citrobacter* spp. (36.8 %), *Enterobacter* spp. (23.7 %), *Klebsiella* spp. (17.1 %), *E. coli* (21.1 %), *Enterococcus* spp. (14.5 %), *Serratia* spp. (2.6%), * staphylococcus* spp. (2.6 %), and *proteus* spp. (3.9 %). Similarly, in a study done by Islam et al., *E. coli* (60 %), *Klebsiella* spp. (40 %), *Enterobacter* spp. (20 %), *Pseudomonas* spp. (70 %), *Proteus* spp. (10 %), *Staphylococcus* spp. (40 %) were found [10].

In our study, all the tap water samples had acceptable pH. Similar types of results were also found by Chaidez et al. [28] and Abed and Alwakeel [14]. The average temperature was between 28.7 °C and 29.38 °C in the study by Chaidez et al. [28], which was between 11 °C  and 26 °C in our study. The temperature of water is influenced by the temperature of the environment.

Finally, from the literatures we have reviewed, it can be concluded that bacteriological contamination of drinking water is a significant problem not only in Nepal but also in other south Asian countries and other parts of the world like Sudan [35], Makkah al-Mokaarama [36], Egypt [37], Canada [38], and Mexico [28].

### Limitations of the study

Since this study was conducted in a low-income country, with limited resources, we could not process large numbers of the samples. Further, we could not use molecular technology for the identification of the bacteria isolated. The use of large numbers of the samples in the study would have generated more significant results. And the molecular methods are the best methods for the proper identification of the organisms. We could not include the detection of pathogens like pathogenic bacteria (*Salmonella* spp., *Vibrio cholerae*, etc.), viruses, fungi, and parasites (protozoa and helminths) in our study. These microorganisms may be present in the water and may be responsible for large numbers of infections, but for their detection, more effort and additional resources are needed. In different seasons, the microbial flora presenting in the water may differ due to different environmental conditions of the surrounding. But in this study, we could not study the seasonal variation of the microorganisms present in the water, as for this more samples needed to be processed and for which we did not have sufficient resources.

## Conclusions

One hundred percent of the municipal tap water samples and most of the bottled drinking water samples distributed in Dharan were found to be contaminated with indicator organisms in counts exceeding WHO standards. The findings of our study suggest that comparatively, the bottled drinking water may be safer (than tap water) to drink.

## Abbreviations

BEA, bile esculin agar; BPKIHS, B. P. Koirala Institute of Health Sciences; EMB, eosine methylene blue agar; FCC, fecal coliform count; FSC, fecal streptococcal count; GPR, gram-positive rods; HPC, heterotrophic plate count; MEA, m-endo agar les; ml, milliliter; MS, Microsoft; PCA, plate count agar; SD, standard deviation; SPSS, statistical package for social sciences; TCC, total coliform count; TPC, total plate count; WHO, World Health Organization
